# The differential impact of oral poliovirus vaccine formulation choices on serotype-specific population immunity to poliovirus transmission

**DOI:** 10.1186/s12879-015-1116-4

**Published:** 2015-09-17

**Authors:** Kimberly M. Thompson, Radboud J. Duintjer Tebbens

**Affiliations:** Kid Risk, Inc., 10524 Moss Park Rd., Ste. 204-364, Orlando, FL 32832 USA

**Keywords:** Polio, Eradication, Risk management, OPV, Vaccine choice

## Abstract

**Background:**

Prior analyses demonstrated the need for some countries and the Global Polio Eradication Initiative (GPEI) to conduct additional supplemental immunization activities (SIAs) with trivalent oral poliovirus vaccine (tOPV) prior to globally-coordinated cessation of all serotype 2-containing OPV (OPV2 cessation) to prevent the creation of serotype 2 circulating vaccine-derived poliovirus (cVDPV2) outbreaks after OPV2 cessation. The GPEI continues to focus on achieving and ensuring interruption of wild poliovirus serotype 1 (WPV1) and making vaccine choices that prioritize bivalent OPV (bOPV) for SIAs, nominally to increase population immunity to serotype 1, despite an aggressive timeline for OPV2 cessation.

**Methods:**

We use an existing dynamic poliovirus transmission model of northwest Nigeria and an integrated global model for long-term poliovirus risk management to explore the impact of tOPV vs. bOPV vaccine choices on population immunity and cVDPV2 risks.

**Results:**

Using tOPV instead of bOPV for SIAs leads to a minimal decrease in population immunity to transmission of serotypes 1 and 3 polioviruses, but a significantly higher population immunity to transmission of serotype 2 polioviruses. Failure to use tOPV in enough SIAs results in cVDPV2 emergence after OPV2 cessation in both the northwest Nigeria model and the global model. Despite perceptions to the contrary, prioritizing the use of bOPV over tOPV prior to OPV2 cessation does not significantly improve serotype 1 population immunity to transmission.

**Conclusions:**

Immunization leaders need to focus on all three poliovirus serotypes to appropriately manage the risks of OPV cessation in the polio endgame. Focusing on population immunity to transmission to interrupt WPV1 transmission and manage pre-OPV cessation risks of cVDPVs, all countries performing poliovirus SIAs should use tOPV up until the time of OPV2 cessation, after which time they should continue to use the OPV vaccine formulation with all remaining serotypes until coordinated global cessation of those serotypes.

## Background

National immunization programs and the Global Polio Eradication Initiative (GPEI) continue to manage risks associated with the polio endgame, including the risks of using oral poliovirus vaccine (OPV) to end all transmission of wild polioviruses (WPVs). The GPEI currently plans to globally-coordinate cessation of all serotype-2 containing OPV (i.e., OPV2 cessation) according to an aggressive timeline with a target date of April 2016 [1]. Prior modeling studies demonstrated the need for sufficient supplemental immunization activities (SIAs) with OPV prior to globally-coordinated OPV cessation to prevent the creation of circulating vaccine-derived poliovirus (cVDPV) outbreaks after OPV cessation [2–6]. The planned addition of a single dose of inactivated poliovirus vaccine (IPV) given at the time of the third trivalent (tOPV) dose of routine immunization (RI) for countries currently using OPV-only for immunization [1] appears to offer only marginal benefits for areas most at risk of cVDPVs after OPV cessation [3]. While clinical trials suggest that IPV boosts intestinal immunity more than OPV in individuals with prior immunity induced by a live poliovirus (LPV, i.e., WPV, OPV, cVDPV, or any OPV-related viruses) [7, 8], models consistent with this observation at the individual level show a minimal impact of IPV on immunity to poliovirus transmission at the population level [3, 5, 6]. This occurs because while IPV protects individual vaccine recipients from developing paralysis if they become infected with an LPV, IPV use leads to relatively little impact on fecal-oral poliovirus transmission in previously susceptible individuals and therefore in populations with conditions conducive to fecal-oral poliovirus transmission. Conditions associated with relatively higher-income countries (i.e., low population density, good hygiene, relatively greater role of oropharyngeal transmission) [9] increase the relative impact of IPV on population immunity to poliovirus transmission, but the minimal conditions for IPV to provide sufficient population immunity to prevent poliovirus transmission remain uncertain. The recent experience with WPV1 transmission in Israel despite high RI coverage with IPV provided valuable context [10].

Following a shift in GPEI policy that began in the mid-2000s from exclusive use of tOPV to the use of monovalent OPV (mOPV) in SIAs in endemic countries with the expectation that using mOPV serotype 1 (mOPV1) would soon stop wild poliovirus (WPV) serotype 1 (WPV1) transmission in endemic areas [11], OPV formulation vaccine choices for SIAs became an important factor in the overall population immunity to transmission for each of the three poliovirus serotypes. The GPEI and some statistical analyses [12, 13] define population immunity as vaccine-induced immunity to disease among children under 5 years of age with non-polio acute flaccid paralysis. This characterization of population immunity to disease does not account for immunity derived from exposure to LPVs in the environment and it ignores the contributions to transmission of individuals of all ages immune to disease who can still participate in asymptomatic transmission [14]. The resulting vaccine choices for SIAs focus on the premise that competition between serotypes in tOPV in individuals leads to “vaccine failure” for serotypes 1 and 3 due to relatively lower take rates for individuals receiving tOPV than for those receiving mOPV [11, 13, 15]. In contrast with this individual vaccine-induced characterization of population immunity, our characterization of population immunity to poliovirus transmission uses a dynamic disease model to characterize immunity to poliovirus transmission of all three serotypes for all individuals in the entire population based on their exposure history to vaccines and circulating LPVs and focuses on the level of population immunity needed to stop viral transmission [9, 14].

Despite high expectations, the single serotype (mOPV1) strategy failed to achieve WPV1 interruption and led to outbreaks with serotype 3 WPV (WPV3), which then motivated the introduction of serotype 3 monovalent OPV (mOPV3) followed by bivalent OPV (bOPV, serotypes 1 and 3) for SIAs. Recent modeling suggested that the strategy pursued (i.e., the introduction of mOPVs then bOPV) delayed the interruption of WPVs in India [5]. Continued failures to achieve and maintain high population immunity to transmission delay eradication and allow WPV importations and cVDPV emergences and importations to cause outbreaks [16]. Despite the somewhat lower relative take rates for serotypes 1 and 3 characterized as “vaccine failure” for tOPV, the experience and models of the last endemic areas in which clusters of under-vaccinated children repeatedly miss immunizations and sustain WPV transmission suggests that “failure to vaccinate” represents the fundamental problem [4–6, 17], confirmed for Nigeria recently by a subsequent independent analysis [18]. For example, despite the large numbers of SIAs conducted each year in northwest Nigeria, between 2010 and 2014, 47 % (121/256) of all confirmed WPV1, WPV3, and cVDPV2 cases reported receipt of 2 OPV doses or fewer, which may include some heterotypic OPV doses (e.g., bOPV doses received by patients paralyzed by cVDPV2).

Modeling particularly indicates the need for more tOPV SIAs to prevent serotype 2 cVDPV (cVDPV2) outbreaks after OPV2 cessation [4]. The GPEI continues to focus on achieving and ensuring interruption of WPV1 transmission and making vaccine choices that prioritize the use of bOPV for SIAs, nominally to increase population immunity to disease for serotype 1, despite an aggressive timeline for OPV2 cessation. However, achieving and maintaining polio eradication requires permanently stopping and preventing transmission, which requires a focus on population immunity to transmission, not a focus on population immunity to disease. This paper aims to explore the trade-offs in population immunity to transmission for the three poliovirus serotypes for tOPV or bOPV use in SIAs.

## Methods

We use an existing differential equation-based dynamic poliovirus transmission and OPV evolution model [9] (i.e., the DEB model) to characterize the impacts of different vaccine choices for SIAs in northwest Nigeria [4, 6, 17, 19] and an integrated global model of long-term poliovirus risk management (i.e., the global model) [20] to explore the impacts of tOPV vs. bOPV vaccine choices on global population immunity to poliovirus transmission and cVDPV risks. Briefly, the DEB model dynamically tracks individuals as they move between immunity states because they acquire immunity from maternal antibodies, successful IPV vaccination, successful OPV vaccination or infection due to contact with an OPV vaccine recipient, or other LPV exposure, and lose immunity in the absence of further vaccinations or infections due to waning. We define successful vaccination as receipt of a vaccine that “takes” (i.e., typically approximated by seroconversion as measured in clinical trials), with take rates that depend on the vaccine and setting. We determined situation-specific average per-dose take rates for all available poliovirus vaccines by model calibration within ranges from the literature [9, 21]. In some cases, this included adjustments to account for study limitations (e.g., different settings and vaccines used in clinical trials than in the modeled population, possible interference with maternal antibodies or secondary OPV infections with study results). In the DEB model, cVDPVs emerge when population immunity to transmission becomes so low that OPV-related viruses introduced through RI or SIAs can sustain transmission in the population and evolve to successive reversion stages with increasingly high basic reproduction numbers (R_0_ values) and paralysis-to-infection ratios (PIRs). When the prevalence in the last of 20 reversion stages chosen to adequately represent the OPV evolution process [9, 22] exceeds a given transmission threshold, then fully-reverted VDPVs with assumed equal R_0_ and PIR as homotypic WPV circulate in the population and a cVDPV outbreak can occur.

We characterize population immunity to poliovirus transmission in two different but related ways [4, 6]. The mixing-adjusted effective immunity proportion (EIPM) represents the proportion of immune individuals in a population, weighted by the relative potential contribution to transmission for their immunity state and the extent to which they mix with other age groups or connected subpopulations. If EIPM remains above its threshold EIP* = 1/(1-R_0_), then transmission eventually stops, while for EIPM < EIP* transmission can continue and imported viruses can establish transmission. However, because EIP* depends on R_0_, which changes over time and varies by serotype and setting, for this analysis we focus on the mixing-adjusted net reproduction number (R_n_), which equals R_0_ × (1-EIP) and represents the average number of secondary infections generated by a single infectious individual, taking into account mixing between age groups and subpopulations and the relative potential contribution to transmission of all individuals in the population. The threshold (R_n_*) equals 1 for any R_0_, serotype, setting, or point in time [4, 19], such that if R_n_ > 1, then each new infection generates at least one new infection and transmission of existing or imported poliovirus can continue, but if R_n_ < 1 for a long enough period of time then transmission eventually dies out.

To explore SIA vaccine choices in the northwest Nigeria DEB model, we adopt all inputs from the most recent model update [4, 19]. This includes assumed continuation of the status quo of 9 annual SIAs until globally-coordinated cessation of serotype 1 and serotype 3-containing OPV (OPV13 cessation) on April 1, 2019. The model further assumes OPV2 cessation on April 1, 2016, which switches RI and SIAs from tOPV to bOPV. Given very low RI coverage (i.e., of 26.4 %, 22.2 %, 18.1 %, and 13.9 % with dose 0 (i.e., birth), 1, 2, and 3, respectively as of 2013) [23], uncertainty about timing, and scale of IPV use going forward, we previously demonstrated very limited impact of IPV on population immunity and cVDPV risks in settings like northwest Nigeria [3, 5, 6]. Consequently, we do not include IPV use in the northwest Nigeria DEB model, although Nigeria began introducing it in SIAs in some limited areas. We report R_n_ as a function of time for different options that use tOPV for between 0 and 9 of the annual SIAs between January 1, 2015 and OPV2 cessation. To spread out the impact of tOPV SIAs over the year while concentrating as many tOPV SIAs as possible in the months before OPV2 cessation on April 1, 2016, we change successive SIAs from bOPV to tOPV in the following order: March, November, August, January, May, December, June, September, April. For example, the option of 3 annual tOPV SIAs implies tOPV use during the March, November, and August SIAs.

The global model divides the world into 710 subpopulations of approximately 10 million people in 2013 and uses the DEB model to track infections and population immunity to transmission in each subpopulation [20]. The global model groups each subpopulation into 9 global regions consisting of variable numbers of epidemiological blocks that in turn consist of 10 subpopulations of equal size to simulate random exportations from subpopulations to other subpopulations in the same block (i.e., representing 96 % of all exportations) or other blocks (i.e., representing 4 % of all exportations, including 3.5 % within the same region and 0.5 % elsewhere). As in the DEB model, cVDPVs can emerge endogenously if population immunity to transmission becomes low enough as long as OPV virus from immunization (including RI) or importations transmit in any subpopulation. The global model characterizes other long-term risks after OPV cessation stochastically, but given our focus on short term population immunity to transmission we ignore those risks in this analysis. The subpopulations in the global model reflect conditions related to poliovirus transmission and vaccination similar to real conditions that exist throughout the world and consistent with available global data [24], but at a more abstract level amenable to our global mixing characterization and simplification of the complex RI and SIA histories in each country [9, 20, 21]. The global model includes 4 blocks with conditions like the last 4 global reservoirs of indigenous WPV1 and WPV3 transmission, which each include an under-vaccinated subpopulation. These 4 subpopulations sustain WPV1 and WPV3 the longest in the model, and because of their very low RI coverage and poor SIA quality they also represent the highest risk areas of cVDPV emergence after OPV cessation.

With respect to SIAs, the global model assumes that blocks increase the annual SIA frequency by one per year each year until they eliminate all indigenous WPV transmission. After elimination of all indigenous WPVs from a block and in the absence of any detected outbreaks, subpopulations conduct between 0 and 6 annual preventive SIAs, depending on their RI coverage with 3 or more non-birth doses (POL3) and R_0_ [20]. For reference, Table 1 provides the specific SIA schedule assumptions. The global model assumes that between 2010 and January 1, 2015, 2–5 annual SIAs in populations that conduct more than 1 annual SIA use bOPV and the remainder use tOPV (Table 1). We assume tOPV intensification starts on January 1, 2015 and switches between 1 and 2 annual SIAs from bOPV to tOPV until OPV2 cessation. We consider the implications of tOPV intensification on all three serotypes by reporting the R_n_ of all three serotypes at the time of OPV2 cessation with or without tOPV intensification.

**Table 1:** Planned, preventive (pSIA) SIA schedules used in the global model before and after OPV2 cessation in OPV-using blocks after interruption of indigenous wild poliovirus transmission in each block (based on Duintjer Tebbens et al. (2015) [20])

Time period	RI coverage (POL3)	SIA schedule showing: vaccine (day(s) of year)
Before tOPV intensification on January 1, 2015	0.05 or 0.1	tOPV (0, 40); bOPV (80, 140, 240, 300)
	0.3	tOPV (0, 40); bOPV (80, 140, 240)
	0.6 (R_0_ ≤ 10)	tOPV (0); bOPV (60, 120)
	0.6 (R_0_ > 10)	tOPV (0, 40); bOPV (80, 140, 240)
	0.9	tOPV (0)
	0.98 (R_0_ ≤ 10)	No SIAs
	0.98 (R_0_ > 10)	tOPV (0)
During tOPV intensification (January 1, 2015 to April 1, 2016)	0.05 or 0.1	tOPV (0, 40, 80, 300); bOPV (140, 240)
	0.3	tOPV (0, 40, 80); bOPV (140, 240)
	0.6 (R_0_ ≤ 10)	tOPV (0, 60); bOPV (120)
	0.6 (R_0_ > 10)	tOPV (0, 40, 80); bOPV (140, 240)
	0.9	tOPV (0)
	0.98 (R_0_ ≤ 10)	No SIAs
	0.98 (R_0_ > 10)	tOPV (0)
After tOPV intensification (April 1, 2016 to OPV13 cessation)	0.05 or 0.1	bOPV (0, 40, 80, 140, 240, 300)
	0.3	bOPV (0, 40, 80, 140, 240)
	0.6 (R_0_ ≤ 10)	bOPV (0, 60, 120)
	0.6 (R_0_ > 10)	bOPV (0, 40, 80, 140, 240)
	0.9	bOPV (0)
	0.98 (R_0_ ≤ 10)	No SIAs
	0.98 (R_0_ > 10)	bOPV (0)

We further explore the option of simultaneously coordinating cessation of all 3 OPV serotypes (OPV123 cessation) on April 1, 2019. Specifically, we compare the R_n_ of each of three serotypes on April 1, 2019 for an option of continued tOPV intensification (i.e., continued use of both tOPV and bOPV for SIAs, as specified in Table 1) until OPV123 cessation with the corresponding R_n_ for an option of exclusive tOPV use for RI and SIAs from January 1, 2017 until OPV123 cessation. We focus the comparison on 165 subpopulations affected by tOPV intensification, which includes all subpopulations with a POL3 of less than 0.9. For all options, the global model assumes that subpopulations that used OPV-only as of 2013 add a single IPV dose co-administered with the third non-birth OPV RI dose on January 1, 2015, consistent with the current plan [1].

**Fig. 1:**
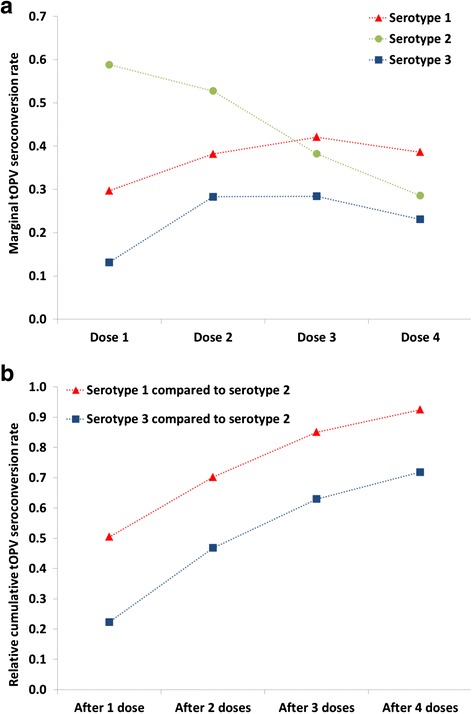
tOPV response to the three serotypes, by number of doses, in a clinical trial in Brazil, 1989[30]. **a** Marginal seroconversion rates, defined as the incremental number of children seroconverting after each dose, divided by the number of children that did not yet seroconvert prior to the dose. **b** Relative cumulative tOPV seroconversion rates, defined as cumulative seroconversion rate for the given serotype after the given number of doses, divided by cumulative seroconversion rate for serotype 2 after the same number of doses

Table 2 summarizes our assumed take rates in the DEB and global models along with data from the only published clinical trials that directly compared current bOPV and tOPV vaccines [25, 26]. The clinical trial conducted in three sites in Central and Southern India reports seroconversion rates for all three serotypes among newborn children vaccinated with tOPV or bOPV at birth and again at 30 days [25]. Table 2 reports average per-dose take rates for tOPV and bOPV, calculated from the cumulative seroconversion rates after 2 doses (CS2) as 1-(1-CS2)^1/2^. The seroconversion rate observed for serotype 2 in the bOPV arm of the trial may reflect a small heterologous serological response, or more likely represents secondary exposure to serotype 2 OPV virus given the intensity OPV exposure in India [27]. The study results translate into a relative reduction of approximately 35 % in the average per-dose individual take rate for tOPV compared to bOPV after two doses (both administered at an age of some partially reduced susceptibility due to maternal antibodies) [28]. However, a recent study that compared bOPV, mOPV1, and tOPV in different schedules in Bangladesh suggests that the difference becomes smaller with subsequent doses, because serotype 2-interference with serotype 1 and 3 seroconversion decreases as individuals become better protected to serotype 2 [26]. For a standard schedule of three doses at 6, 10, and 14 weeks of age, the reduction in the average per-dose individual seroconversion rates for serotypes 1 and 3 (calculated from the cumulative seroconversion rates after 3 doses (CS3) assuming 1-(1-CS3)^1/3^) amounted to only approximately 20 % for tOPV compared to bOPV. Figure 1 summarizes the results from a 1989 Brazil tOPV seroconversion study [29] that confirms that few children seroconvert to serotypes 1 and 3 after the first dose, while more than half seroconvert to serotype 2 (Fig. 1a). However, once the majority of children acquire immunity to serotype 2 after the first dose, serotype interference diminishes, resulting in increased serotype 1 and 3 seroconversion for subsequent doses. Consequently, the differences in cumulative seroconversion rates between serotype 3 and serotype 2 decrease with each successive dose, and those between serotype 1 and serotype 2 almost disappear altogether after 4 doses (Fig. 1b). While the numerical results will differ in places other than Brazil due to environmental and other factors [30], we should similarly expect increased serotype 1 and 3 seroconversion with successive tOPV doses as children develop serotype 2 immunity. This means that for populations, which include a mixture of individuals with different ages and immunization and exposure histories, assumptions about the overall take rate of the different serotypes must recognize that only a small fraction of tOPV SIA recipients did not previously seroconvert to serotype 2. Thus, the receipt of bOPV as a first dose instead of tOPV as a first dose for bOPV SIAs only leads to serotype 1 or 3 seroconversion instead of serotype 2 seroconversion for a relatively small fraction of the population.

**Table 2:** Serotype-specific average per-dose take rates for tOPV and bOPV determined in the clinical trials that compared both vaccines, and assumed in the DEB and global models

Setting	tOPV serotype			bOPV serotype		
	1	2	3	1	2	3
Clinical trials						
Central and Southern India [25], 2 doses at 0 and 30 days of age	0.39	0.70	0.31	0.62	0.06	0.49
Bangladesh [26], 3-doses at 6, 10, and 14 weeks of age)	0.57	0.65	0.51	0.70	0.06	0.65
Calibrated DEB model within ranges from literature [17, 21]						
Northwest Nigeria [4, 6, 9, 17, 19]	0.45	0.70	0.35	0.54	0	0.54
Northern India [5, 9, 17, 19]	0.35	0.60	0.27	0.42	0	0.42
Global model assumptions based on calibrated DEB model [20]						
Lowest tier (e.g., Northern India)	0.35	0.60	0.27	0.42	0	0.42
Second tier (e.g., Northern Pakistan)	0.40	0.65	0.32	0.50	0	0.50
Third tier (e.g., Northwest Nigeria)	0.45	0.70	0.35	0.54	0	0.54
Fourth tier (e.g., Brazil)	0.50	0.72	0.40	0.60	0	0.60
Fifth tier (e.g., Philippines, Turkey)	0.55	0.73	0.45	0.70	0	0.70
Sixth tier (e.g., Russia, middle-income China)	0.60	0.74	0.50	0.75	0	0.75
Seventh tier (e.g., upper-income China, Israel)	0.65	0.75	0.55	0.80	0	0.80

Taking into account the average effect on take of multiple tOPV doses, our model assumes closer average per-dose take rates for serotypes 1 and 3 bOPV and tOPV than observed after 2 doses for very young children in the controlled trial in India (Table 2) [25], but similar relative average per-dose take rates as found in Bangladesh after 3 bOPV vs. tOPV doses [26]. For the northwest Nigeria DEB model, the estimates in Table 2 produce serotype-specific incidence results consistent with the evidence [4, 6, 9, 17, 19]. For the integrated global model, we extrapolated from the northwest Nigeria model and models from other situations, including northern India [5, 9, 17, 19], to assign take rate tiers for different blocks. To explore the impact of differences between tOPV and bOPV serotype 1 and 3 takes rates as large as reported after 2 doses in the clinical trial in India [25], we perform a sensitivity analysis that uses the observed average per-dose rates as estimated from these data in Table 2 instead of our calibrated model estimates.

## Results

**Fig. 2:**
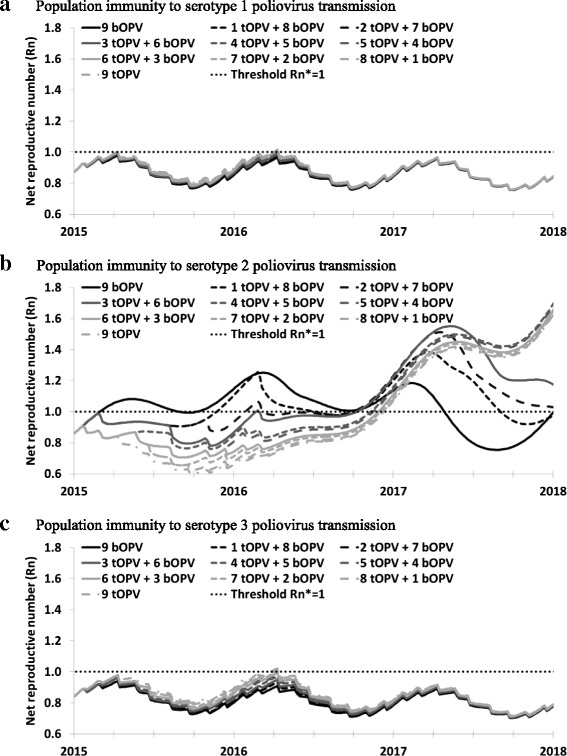
Population immunity to transmission in northwest Nigeria for all 3 serotypes and different annual numbers of bOPV and tOPV SIAs. **a** Population immunity to serotype 1 poliovirus transmission. **b** Population immunity to serotype 2 poliovirus transmission. **c** Population immunity to serotype 3 poliovirus transmission

Figure 2 shows population immunity to transmission of each serotype between 2015 and 2018 for different SIA vaccine choices in the northwest Nigeria model [4, 6, 9, 17, 19]. Higher R_n_ values indicate more potential transmission per new infection and they correspond to lower population immunity (i.e., higher risk of transmission). Despite the assumed lower average per-dose serotype 1 and 3 take rates for tOPV compared to bOPV (Table 2), the high number of SIAs containing serotype 1 and 3 OPV results in very similar population immunity to transmission for serotype 1 and 3 regardless of the number of tOPV and bOPV SIAs (note the similarity of all curves Fig. 1a, c). Consequently, as long as northwest Nigeria maintains the same quality and frequency of SIAs [4], serotype 1 and 3 population immunity to transmission remain far enough below the threshold to minimize the risk of re-established WPV transmission from imported WPV or continued undetected WPV circulation [19], regardless of the proportion of the SIAs using tOPV or bOPV. In contrast, given that bOPV does not provide any immunity against serotype 2, the number of tOPV SIAs greatly influences population immunity to serotype 2 poliovirus transmission (Fig. 1b). Specifically, for 3 or fewer annual tOPV SIAs between January 1, 2015 and the time of planned OPV2 cessation, population immunity to transmission at the time of OPV2 cessation does not become high enough to prevent cVDPV2 emergence after OPV2 cessation and/or to interrupt cVDPV2 transmission before OPV2 cessation. Due to the cVDPV2 outbreak that occurs with 3 or fewer annual tOPV SIAs, during 2017 the resulting viral circulation leads R_n_ to decrease (i.e., population immunity to transmission increases due to cVDPV2 transmission) despite no OPV2 use, which represents a failure associated with OPV2 cessation that will require outbreak response. With 4 or more annual tOPV SIAs, R_n_ continues to increase after OPV2 cessation because OPV2 use stops and all serotype 2 LPV transmission dies out during 2016. Prevention of cVDPV2s clearly represents the better option for achieving high population immunity to transmission for serotype 2 from a health perspective and in the context of managing global cVDPV2 risks, and this analysis demonstrates that tOPV vs. bOPV vaccine choices matter.

**Fig. 3:**
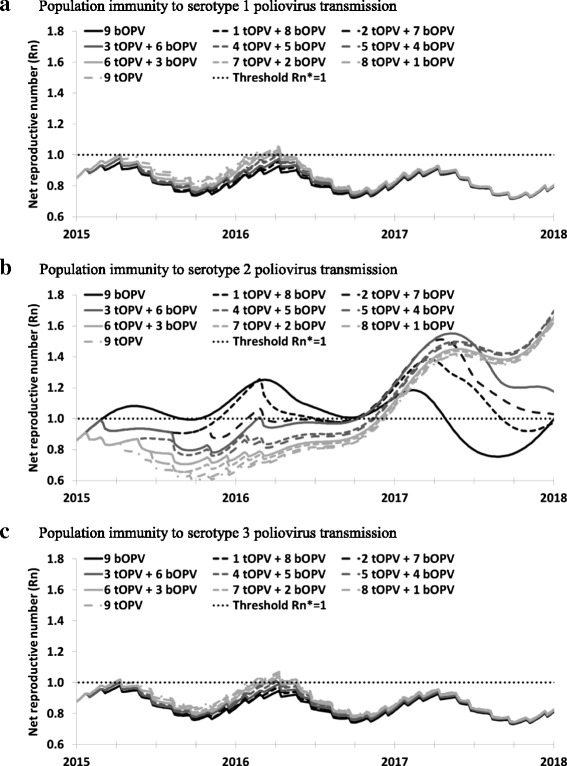
Same model result as in Fig. 2, but with tOPV and bOPV take rates calculated directly from cumulative 2-dose seroconversion estimates reported by Sutter et al. (2010)[27] (see Table 1). **a** Population immunity to serotype 1 poliovirus transmission. **b** Population immunity to serotype 2 poliovirus transmission. **c** Population immunity to serotype 3 poliovirus transmission

Figure 3 shows the population immunity results if the difference between average per-dose tOPV and bOPV take rates for serotypes 1 and 3 become as large as observed in the clinical trial in India after 2 doses (Table 2), thus assuming the entire population behaves like the limited population in the trial [25]. For serotype 2, the results do not change at all, because the serotype 2 tOPV take rate remains unchanged (Fig. 3b). For serotypes 1 and 3, the gap between the population immunity to transmission curves during 2015 and 2016 in Fig. 3a, c increases somewhat compared to those in Fig. 2a, c. However, the difference in population immunity to transmission remains significantly smaller than for serotype 2. Moreover, population immunity to transmission remains high enough to prevent re-established WPV1 or WPV3 transmission during 2015 and 2016. After OPV2 cessation, the curves become similar again due to bOPV-only use for all options, which prevent cVDPVs of serotypes 1 and 3 after OPV13 cessation (not shown).

**Fig. 4:**
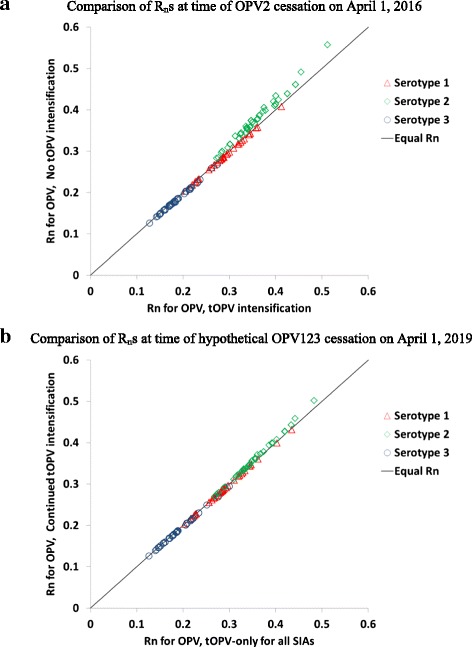
Net reproduction number (R_n_) for OPV of each serotype for different SIA vaccine choices in 165 subpopulations affected by tOPV intensification in the global model [21] . **a** Comparison of R_n_s at time of OPV2 cessation on April 1, 2016. **b** Comparison of R_n_s at time of hypothetical OPV123 cessation on April 1, 2019

Figure 4 shows the results for different SIA vaccine choices in the global model [20]. The results confirm the observations from northwest Nigeria on a global scale. Comparison of options with more tOPV use to options with more bOPV use until OPV2 cessation (i.e., No tOPV intensification vs. tOPV intensification) shows minimal impacts on population immunity to transmission of OPV of serotypes 1 and 3, with the R_n_ values in all subpopulations remaining very close to the line indicating no difference between the two policies (Fig. 4a). However, for OPV serotype 2, Fig. 4a shows higher R_n_ values in all subpopulations without tOPV intensification, implying a higher risk of cVDPV2 outbreaks after OPV2 cessation. Without tOPV intensification, although the R_n_ values remain below 1 at OPV2 cessation, they increase after OPV2 cessation, allowing transmission of increasingly more transmissible OPV-related viruses. This leads to cVDPV2 outbreaks after OPV2 cessation in the global model without tOPV intensification that do not occur with tOPV intensification [20]. Fig. 4b shows that even exclusive tOPV use for all SIAs does not significantly reduce population immunity to transmission of OPV serotypes 1 and 3, while it results in a further marginal increase in population immunity to transmission of OPV serotype 2 compared to continued tOPV intensification. Figure 4b assumes hypothetical simultaneous cessation of all 3 OPV serotypes (i.e., OPV123 cessation) in 2019 and does not lead to cVDPV outbreaks of any type after OPV123 cessation for either of the options. The absence of cVDPV1 and cVDPV3 outbreaks relates to the relatively lower R_0_ values for OPV serotypes 1 and 3 and their slower evolution to fully-reverted VDPVs [9, 17, 22], which results in faster die-out of serotypes 1 and 3 OPV-related viruses compared to serotype 2 OPV-related viruses for any given population immunity level. However, as with OPV2 cessation, continued SIAs with OPV containing serotypes 1 and 3 up until OPV13 cessation remain necessary in populations with low RI coverage to prevent subsequent cVDPV1 and cVDPV3 outbreaks.

## Discussion

Misplaced focus on vaccine failure and on vaccine-induced population immunity to disease [11, 13] continues to allow insufficient focus on the failure to vaccinate and the importance of managing population immunity to transmission of all three serotypes [2, 14, 16, 17]. Failing to achieve and maintain high population immunity to transmission leads to outbreaks and delays both WPV eradication and successful OPV cessation, which increases the overall costs of polio eradication. The complexity of simultaneously managing all 3 poliovirus serotypes requires sufficient use of poliovirus vaccine containing all 3 serotypes (i.e., tOPV or IPV). For countries with conditions conducive to intense transmission (e.g., high R0, relatively poor hygiene, and frequent fecal-oral contacts), even high coverage RI with IPV - only may prove insufficient to prevent transmission [10, 16]. Although the relatively lower individual take rates for serotypes 1 and 3 for tOPV compared to bOPV may suggest a benefit associated with preferentially using bOPV for SIAs [25, 26], our results demonstrate the importance of the serotype 2 component in tOPV and the relatively small impact on population immunity to transmission of using bOPV for SIAs instead of tOPV. Moreover, with repeated tOPV immunizations, recipients develop serotype 2 immunity, which reduces serotype interference and makes subsequent tOPV doses *de facto* bOPV doses. Immunization leaders need to focus on all three poliovirus serotypes to appropriately manage the risks of both WPV eradication and OPV cessation in the polio endgame. Our analyses suggest that all countries performing polio SIAs should use tOPV up until the time of OPV2 cessation, after which time they should continue to use the OPV vaccine formulation with all remaining serotypes until coordinated global cessation of those serotypes as they manage population immunity to transmission.

While our model insights remain very robust to a range of assumptions about the differential impact of tOPV and bOPV on serotype 1 and 3 take rates, we note several limitations. First, the model does not explicitly characterize serotype interference but instead focuses on average per-dose take rates. However, using first-dose take rates instead would artificially decrease the impact of tOPV on serotype 1 and 3 poliovirus transmission, particularly in the context of frequent immunization contacts associated with either good RI coverage, or frequent SIAs, or both. The large difference associated with first-dose take rates would only apply in a situation in which children receive only one effective dose. Second, our model relies on sets of generic model inputs based on an expert literature review process [22, 28, 31] that fits the evidence across a wide range of situations [9, 17] but that does not preclude the possibility that other combinations of generic model inputs may produce results overall consistent with the evidence. For example, the kinetics of waning remain uncertain and intestinal immunity may wane more steeply after 5 or more years [32] than assumed in our model [17] based on the limited evidence [28, 31, 33], which would result in more cVDPV emergences and cases both before and after OPV2 cessation. Thus, further empirical evidence and study of model uncertainties would further help inform decisions. Using different take rates (Fig. 3) than those based on the model calibration process led to some changes in the historical fit, and we did not explore the impact of take rates that vary by dose given that this would add significant complexity to the model (i.e., stratification of the model by dose histories in addition to immunity state, reversion stage, age group, serotype, and waning stage) without good data to support dose-specific take rate estimates in different settings. Nevertheless, the insights with respect to bOPV vs. tOPV in the context of repeated SIAs proved robust to the alternative assumptions about average per-dose take rates vs. the higher serotype interference associated with the first two doses, and we likewise expect robustness of the insights to different potential combinations of generic model inputs that remain consistent with the body of evidence on poliovirus immunity and transmission [28]. Third, the situation in northwest Nigeria or elsewhere may change in the future, particularly related to the confirmed cVDPV2 case reported in May 2015 [34], which should motivate Nigeria to use tOPV for more SIAs. If SIA frequency and/or quality decrease going forward, re-emerging WPV transmission may occur, cVDPV2 transmission may not stop even with four annual tOPV SIAs, or cVDPVs could emerge after OPV cessation. Fourth, for the northwest Nigeria model, we did not model the evolving policies involving IPV SIAs, given their uncertain role in the immunization program. Studies on the impact of IPV SIAs on population immunity to poliovirus transmission of all three serotypes remains a topic of further research. Finally, all limitations associated with the models used in this analysis apply, and we refer to other publications for further discussion of those limitations [9, 20].

## Conclusions

Using tOPV instead of bOPV for SIAs leads to a minimal decrease in population immunity to transmission for serotypes 1 and 3, but a significantly higher population immunity to transmission for serotype 2. Failure to use tOPV in enough SIAs results in cVDPV2 outbreaks after OPV2 cessation in both the northwest Nigeria model and the global model. Immunization leaders need to focus on all three poliovirus serotypes to appropriately manage the risks of OPV cessation in the polio endgame. Focusing on population immunity to transmission to manage pre-OPV cessation risks of cVDPVs, all countries performing poliovirus SIAs should use tOPV up until the time of OPV2 cessation, after which time they should continue to use the OPV vaccine formulation with all remaining serotypes until coordinated global cessation of those serotypes.

## Abbreviations

bOPV: Bivalent oral poliovirus vaccine(serotypes 1 and 3)
cVDPV(1,2,3): Circulating vaccine-derived poliovirus(serotype 1, 2, or 3, respectively)
EIP*: Threshold effective immune proportion
EIPM: Mixing-adjusted effective immune proportion
GPEI: Global Polio Eradication Initiative
IPV: Inactivated poliovirus vaccine
LPV: Live poliovirus
mOPV(1,2,3): Monovalent oral poliovirus vaccine (serotype 1, 2, or 3, respectively)
OPV: Oral poliovirus vaccine
R_0_: Basic reproduction number
R_n_: Mixing-adjusted net reproduction number
RI: Routine immunization
SIA: Supplemental immunization activity
tOPV: Trivalent oral poliovirus vaccine
WPV(1,2,3): Wild poliovirus (serotype 1, 2, or 3, respectively)
